# Synthesis of *Acetobacter xylinum* Bacterial Cellulose Aerogels and Their Effect on the Selected Properties

**DOI:** 10.3390/gels11040272

**Published:** 2025-04-05

**Authors:** Sebnem Sozcu, Jaroslava Frajova, Jakub Wiener, Mohanapriya Venkataraman, Blanka Tomkova, Jiri Militky

**Affiliations:** Department of Material Engineering, Faculty of Textile Engineering, Technical University of Liberec, 46117 Liberec, Czech Republic; jaroslava.frajova@tul.cz (J.F.); jakub.wiener@tul.cz (J.W.); blanka.tomkova@tul.cz (B.T.); jiri.militky@tul.cz (J.M.)

**Keywords:** *Acetobacter xylinum* bacteria, lyophilization, bacterial cellulose aerogel

## Abstract

Bacterial cellulose (BC) synthesized by *Acetobacter xylinum* has gained significant attention due to its unique structural and functional properties. This study focuses on the simple, facile, and cost-effective synthesis of bacterial cellulose films from *Acetobacter xylinum* and evaluates their impact on selected properties. The BC films were prepared through a series of controlled fermentation, purification, and drying processes, optimizing their porosity and structural integrity with different stabilization forms (the BC films supported by polyester nonwoven (PES NW) fabric) by a static culture method keeping with the sustainability. The selected properties like density, porosity, surface roughness, thermal conductivity, and the wetting properties of surfaces are tested. These properties were chosen because they significantly impact the performance of BC aerogels in the potential application of aerogels in biomedical, insulation, and filtration industries. The results indicated that the synthesized BC aerogels exhibit a highly porous network, lightweight structure, and excellent thermal conductivity, making them suitable for advanced material applications. This research highlights the potential of bacterial cellulose aerogels as sustainable (without any additives/chemicals) and high-performance materials, paving the way for further advancements in bio-based aerogels.

## 1. Introduction

Bacterial cellulose (BC) has gained significant attention due to its unique physicochemical properties, making it a highly valuable material in various applications. Unlike plant-derived cellulose, BC is produced by specific bacterial strains, resulting in a highly pure form, free from lignin and hemicellulose. It exhibits exceptional mechanical strength (tensile strength: 200–300 MPa), high crystallinity (60–90%), and superior water-holding capacity (up to 99% water content per unit mass), along with remarkable biocompatibility. These properties make BC suitable for biomedical applications (e.g., wound dressings, tissue engineering), the food industry (as a stabilizer and dietary fiber), packaging (as a biodegradable alternative to plastics), environmental remediation (as an absorbent for pollutants) [1,2,3,4], and insulation applications [5]. However, conventional BC production methods often involve synthetic additives, costly carbon sources, or chemical modifications, which pose environmental and economic concerns. To address these challenges, research has increasingly focused on developing sustainable production strategies that minimize environmental impact while maintaining the high performance of BC.

Recent advancements in the field have demonstrated various approaches to obtaining BC under sustainable conditions. One of the most promising strategies involves utilizing agro-industrial waste and renewable carbon sources, such as fruit peels, molasses, and lignocellulosic biomass, as fermentation substrates. Studies have shown that these alternative carbon sources can effectively support BC biosynthesis while significantly lowering production costs and reducing environmental waste [6]. For instance, researchers have successfully employed waste-based media derived from pineapple peels, coconut water, and corn steep liquor to produce high-yield BC with desirable properties. Additionally, optimizing culture conditions, such as pH, temperature, and aeration, have been explored to enhance BC yield without the need for synthetic additives [7]. Bioreactor-based production methods have also been investigated as an eco-friendly approach to BC synthesis. The use of sustainable bioprocessing techniques, including static and dynamic fermentation, has been explored to maximize productivity and quality. Moreover, some studies have focused on genetic modifications of BC-producing bacteria to improve cellulose synthesis efficiency while maintaining sustainability. Nevertheless, while these advancements are promising, many of the existing approaches still rely on supplementation with chemicals or engineered modifications, which can compromise the sustainability aspect of BC production [8,9].

By adopting a holistic, sustainable methodology, our study focuses on the simple, facile, sustainable, and cost-effective synthesis of bacterial cellulose films from *Acetobacter xylinum* and evaluates their impact on selected properties. The BC films were prepared through a series of controlled fermentation, purification, and drying processes, optimizing their porosity and structural integrity with different stabilization forms (the BC films supported by Polyester nonwoven (PES NW) fabric after synthesized) by static culture method with keeping the sustainability. The selected properties, like density, porosity, surface roughness, thermal conductivity, and the wetting properties of surfaces, were tested. These properties were chosen because they significantly impact the performance of BC aerogels in the potential application of aerogels in biomedical, insulation, and filtration industries. This research highlights the potential of bacterial cellulose aerogels as sustainable (without any additives/chemicals compared to literature) and high-performance materials, paving the way for further advancements in bio-based aerogels. This study not only demonstrates an eco-friendly alternative to conventional methods but also offers a scalable and industrially relevant approach that aligns with the principles of green chemistry and circular economy. Our findings contribute to the broader goal of sustainable material development, offering a novel perspective on achieving high-quality BC while preserving environmental integrity.

## 2. Results and Discussion

Under stationary growth conditions, *Acetobacter xylinum* synthesizes bacterial cellulose, which forms a film on the surface of the nutrient medium. Various factors, including temperature, duration, and the surface area-to-substrate volume ratio, significantly influence the efficiency of this biosynthesis process. Among these, temperature plays a crucial role in regulating oxygen availability, as both insufficient and excessive oxygen levels negatively impact bacterial cellulose production by affecting microbial activity [10]. Throughout the bacterial cellulose (BC) growth process, periodic temperature fluctuations were observed due to laboratory conditions. These variations are particularly important, as maintaining an optimal temperature is essential for promoting the rapid formation of BC films. Despite these fluctuations, BC films of varying thicknesses and dimensions were successfully synthesized as required.

**Figure 1:**
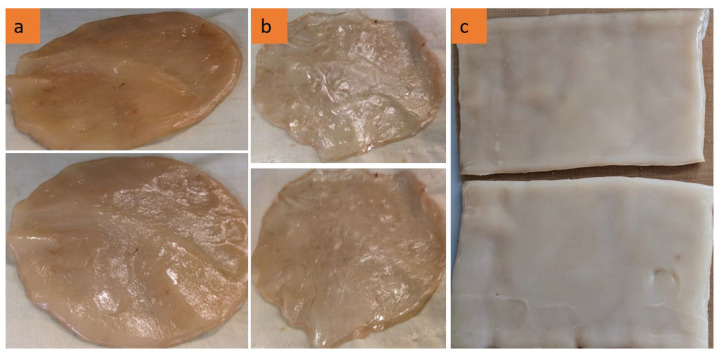
The BC samples were collected following the cultivation procedure. (**a**) Circular and thick structure, (**b**) irregular and thin structure, (**c**) rectangular and processed sheets.

As demonstrated in Figure 1, the morphological variations observed in BC films are directly associated with their respective growth durations. Figure 1a, b depict bacterial cellulose (BC) films cultivated in Petri dishes. Compared to Figure 1a, Figure 1b exhibits increased translucency and reduced structural integrity, indicating an earlier growth stage and the formation of thinner BC films. In contrast, Figure 1a displays a thicker, smoother, and fully developed BC film with a relatively uniform surface, suggesting that it has undergone sufficient growth and fermentation, thereby establishing a well-structured cellulose network. Additionally, Figure 1c presents a BC film that is thicker, denser, and more homogeneous, characterized by a well-defined rectangular shape.

The results validate that BC films can be cultivated into specific shapes and thicknesses utilizing only a tea solution, sugar, and bacterial cells without the necessity of chemical additives. Moreover, the application of a static cultivation method under controlled laboratory conditions highlights the sustainability and cost-effectiveness of this approach.

### 2.1. Geometrical Characterization

Bacterial cellulose (BC) aerogels have garnered significant attention due to their distinctive structural and mechanical properties, positioning them as promising materials for a range of biomedical, environmental, and industrial applications. The geometrical characterization of BC aerogels, encompassing parameters such as dry thickness, dry mass, volume density, porosity, dimensions, and shape, is essential for comprehending their structural integrity and functional performance following lyophilization.

This study utilized various fabrication and freezing methods to examine their impact on the geometric properties of BC aerogels. The samples were fabricated using different supporting structures, including nonwoven (NW) fabric, plastic frames, and liquid nitrogen (LN_2_) pre-freezing techniques. These methodologies significantly influenced the final thickness, density, and porosity of the aerogels, thereby affecting their mechanical and functional properties.

The results summarized in Table 1 provide an overview of the primary geometrical parameters of BC aerogels following lyophilization. The data indicate variations in shape, dimension, porosity, and density, which are dependent on the preparation method. Specifically, pre-freezing with LN_2_ notably enhances both the dry mass and volume density when compared to NW-supported aerogels. Moreover, samples produced with plastic frames exhibit the highest volume density. Notably, the porosity consistently exceeds 98% in all samples, underscoring the lightweight characteristics of BC aerogels.

However, the LN_2_ pre-freezing method (Samples F, G, H)) led to extremely high porosity (~99.97–99.99%), which seems counterintuitive given their high mass and density. This suggests that although they retain more material, they still have a very open and void-rich structure due to ice crystal formation. Despite being expected to have high porosity due to their fibrous structure of samples (A, B, C, D), these samples actually have slightly lower porosity (~98.81–99.54%) than LN_2_ samples. This suggests that while they are airy and lightweight, they do not reach the same level of void space as the LN_2_-treated materials. Sample E has the highest density (360.36 g/cm^3^) while maintaining high porosity (99.87%), showing that the plastic frame restricted expansion, compacting the structure while still retaining significant void space.

Additionally, skeletal density analysis further differentiates the samples. NW-supported aerogels (A, B, C, D) exhibit relatively high skeletal densities (1.01–1.26 g/cm^3^), indicating a more compact solid phase. The plastic frame-supported sample (E) shows the highest skeletal density (1.31 g/cm^3^), reflecting its densified structure due to constrained expansion. Conversely, LN_2_ pre-freezing samples (F, G, H) have extremely low skeletal densities (0.22–0.51 g/cm^3^), confirming their highly porous and lightweight nature despite their high mass. This trend highlights that LN_2_-treated samples prioritize porosity and absorbency, whereas NW-supported and plastic frame-based samples tend to be more structurally compact and rigid.

This result provides insights into the impact of different processing conditions on the structural attributes of BC aerogels, contributing to the optimization of their fabrication for diverse applications.

**Figure 2:**
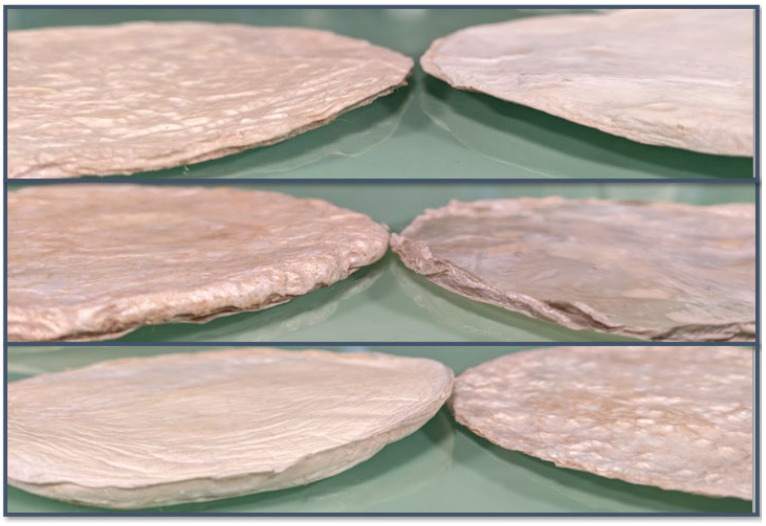
The non-homogeneous surface structure of freeze–dried BCAs (neither form stabilization with NW fabric nor LN_2_ pre-freezing applied to samples).

Figure 2 presents the freeze–dried BCAs without any form of stabilization, with all samples demonstrating identical conditions and configurations. None of the samples shown in the image underwent form stabilization or pre-freezing with LN_2_. These represent the initial state prior to the application of form stabilization or pre-freezing with LN_2_. As depicted in the figure, the BCAs exhibit non-homogeneous surface structures characterized by uneven surfaces with varying thicknesses. Previous studies have compared the visual characteristics of BCA samples dried via oven and lyophilization methods. Vasconcellos et al. and Illa et al. reported that oven-dried samples appeared transparent, whereas freeze–dried samples displayed a foam-like structure resembling Styrofoam while retaining their original shape [11,12]. Consistent with these findings, our results also indicated that freeze–drying preserved the samples’ thickness and foam-like appearance (Figure 2).

**Figure 3:**
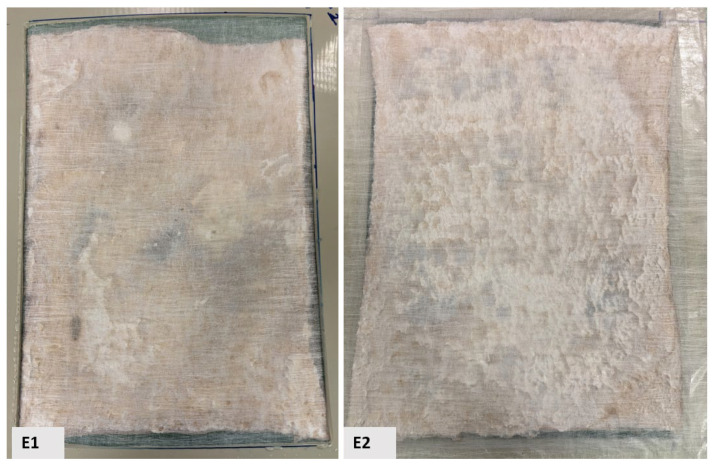
Sample E, a freeze–dried BC film set inside a plastic frame construction [E1 is the sample’s front view; E2 is its back view].

**Figure 4:**
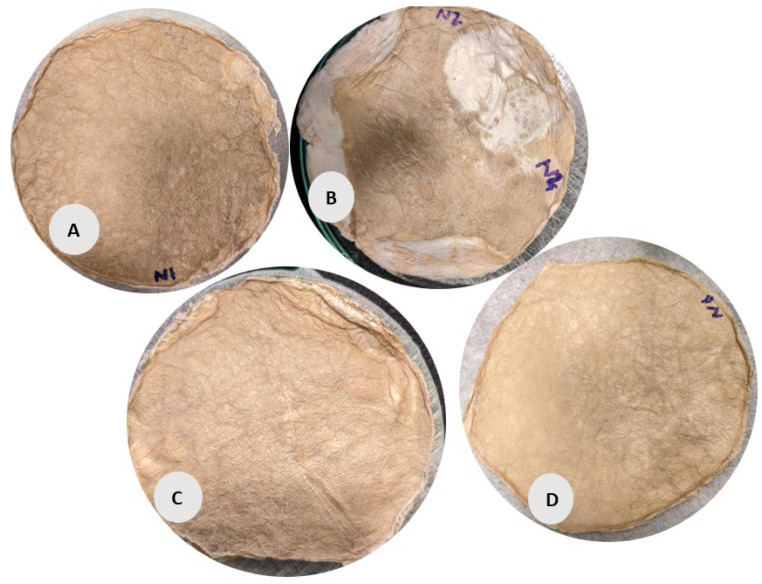
BC films were prepared for form stabilization with the PET NW fabrics. According to the sample description in Table 7. (**A**–**D**) stabilized structure of BC with conventional pre-freezing used before lyophilization.

Alternatively, despite employing various surface stabilization methods on BCAs, achieving uniform surface structures remained elusive. The applied stabilization techniques are illustrated in Figure 3 and Figure 4, respectively. The plastic-framed BC film (sample E) exhibits distinct structural characteristics compared to samples composed of NW fabric, which are lighter and less compact, as well as LN_2_-treated samples, which possess high porosity but reduced density. Its structure is notably compact yet highly porous. The observed surface irregularities on both sides (E1 and E2) in the image suggest potential roughness induced by pre-freezing or drying processes, as evidenced by increased surface crystallization. This roughness may result from conventional freezing methods or physical interactions between the material and the supporting frame.

The circular samples (A, B, C, D) in Figure 4 have a more uniform, smooth surface but with slight irregularities. Samples B and C exhibit noticeable wrinkling and non-uniformity compared to the other circular samples, possibly due to the lower fabric GSM used during its preparation and (slow) conventional pre-freezing. However, Sample C appears more compact with a denser texture, correlating with its relatively higher volume density (48.47 g/cm^3^) and porosity (99.54%). Sample D shows a slightly more porous and less uniform surface than Sample A, aligning with its lower density (34.68 g/cm^3^) according to data given in Table 1. 

**Figure 5:**
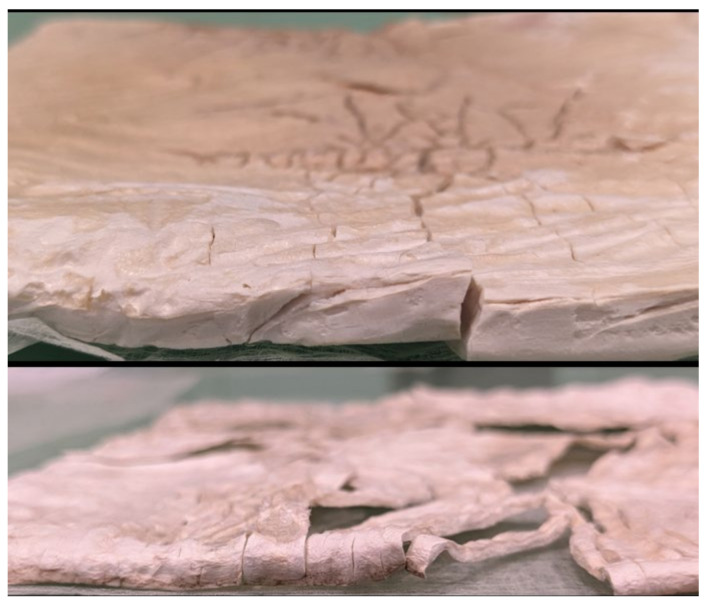
LN_2_ pre-freezing BC films.

The mechanical, thermal, and functional properties of bacterial cellulose (BC) aerogels are directly influenced by their homogenous surface structure, which is significant for a variety of applications. The study investigated pre-freezing conditions due to the absence of significant structural changes in BCA samples following drying in the form-stabilization process. The findings suggest that prolonged storage in a conventional slow-freezing system led to excessive ice crystal formation, resulting in structural damage. A literature review confirmed that both the drying process and the freezing rate prior to lyophilization are critical for achieving homogeneous BCA samples by Zhang et al. and Rang et al. [13,14]. To mitigate these issues, liquid nitrogen (LN_2_) was employed as an alternative to conventional freezing in BC films, as depicted in Figure 5. The images depict structural differences of pre-frozen BC samples with LN_2_ among bacterial cellulose (BC) films subjected to various lyophilization conditions. Both images suggest that these samples have experienced surface damage and fragmentation due to internal stress caused by the rapid sublimation of frozen water. The top image shows a higher-density sample, which remained relatively intact. The bottom image shows a sample that was more brittle due to lower density and internal stress.

Zhang et al. demonstrated that aerogels frozen using liquid nitrogen exhibited a consistent and smooth surface, while those frozen at slower rates developed rough surfaces with streaks due to the formation of ice crystals [13]. Freezing with liquid nitrogen at a rapid rate preserved the aerogels’ intricate porous framework, resulting in a consistent three-dimensional structure composed of fine fibrils ranging from 10–100 nm in diameter. On the other hand, aerogels frozen at slower rates, either at extremely low temperatures (−80 °C) or in conventional freezers (−20 °C), exhibited less porous structures [13]. The research indicated that employing LN_2_ before the freeze–drying process was more effective in forming a highly porous network and thickness in BCAs, yielding a smoother surface with some crack development.

The porosity of the bacterial cellulose structure is greatly influenced by the drying technique applied [12,15]. Methods such as thermal drying under either atmospheric or reduced pressure result in non-porous sheets and clumping of fibers due to the diffusion of water vapor and the formation of hydrogen bonds. On the other hand, freeze–drying and drying with supercritical CO_2_ are more effective methods for maintaining the original three-dimensional network in bacterial cellulose aerogels [12,16,17]. Consistent with the reported cellulose density (1.56 g/cm^3^), it was observed that BCAs show distinct porosity variations when subjected to different pre-freezing techniques, whether gradual or rapid, prior to the drying stage (refer to Table 2, Table 3 and Table 4 ). According to the ANOVA statistical analyses given in Table 2, Table 3 and Table 4, SS, df, MS, F (F-statistic), *p*-value, and F crit (Critical Value) refer to the sum of squares, degrees of freedom, mean square, ratio of the variance between and within groups, probability of results occurring by chance, and threshold for rejecting the null hypothesis, respectively.

Zeng et al. and Al-shamary et al.’s studies proved that the porosity of BCAs dried with freeze–drying by using the liquid nitrogen freezing method have lower F values than conventional pre-freezing methods [18,19]. Since porosity encompasses a wide range of fields, including biomedical engineering, construction, environmental science, sensor technology, and energy systems, their unique characteristics, such as high porosity, low density, and biocompatibility, make them versatile materials with significant potential. Both our ANOVA tests (Table 2) show significant differences among groups (*p*-values < 0.05), indicating that the factors tested (general conditions and LN_2_ freezing porosity) have a statistically significant effect on the results. The first ANOVA (103.17 F-value) shows much stronger differences compared to the second (23.49 F-value). This suggests that the first set of conditions had a greater impact on variation compared to porosity changes in LN_2_ freezing. The F critical values confirm significance. In both cases, the calculated F-value is much higher than the critical threshold, supporting the rejection of the null hypothesis.

Zang et al. and Jiang et al. observed that samples frozen with liquid nitrogen (LN_2_) showed greater density and porosity compared to those frozen using traditional methods, as documented in their research and other studies in the field [13,20]. The conventional pre-freezing BCA ANOVA shows much stronger statistical significance (F = 297.58, *p* < 0.00001) compared to the volume density of LN_2_ freezing (Table 3) (F = 10.16, *p* = 0.0026). The lower within-group MS (12.84 vs. 999.21) in the BCA test indicates more consistency within sample groups. Both tests confirm significant differences between groups, but the BCA conventional pre-freezing data exhibits a much stronger effect size.

A comparison of both ANOVA results reveals that conventional pre-freezing results in significantly greater variations in sample thickness than liquid nitrogen freezing. The higher between-group sum of squares (2.935 vs. 0.497) and the higher F-value (279.36 vs. 23.47) in conventional pre-freezing indicate a more pronounced difference in thickness among sample groups under these conditions (Table 4). This suggests that liquid nitrogen freezing provides more uniform thickness, while conventional pre-freezing leads to substantial variation. Moreover, the within-group mean square values (0.0026 for conventional vs. 0.011 for liquid nitrogen) suggest that the consistency of sample thickness is higher in conventional pre-freezing. However, the extreme statistical significance (*p* < 0.05 in both cases but far lower in conventional pre-freezing) highlights that different freezing methods significantly impact thickness variations.

### 2.2. Scanning Electron Microscopy (SEM) Analysis

**Figure 6:**
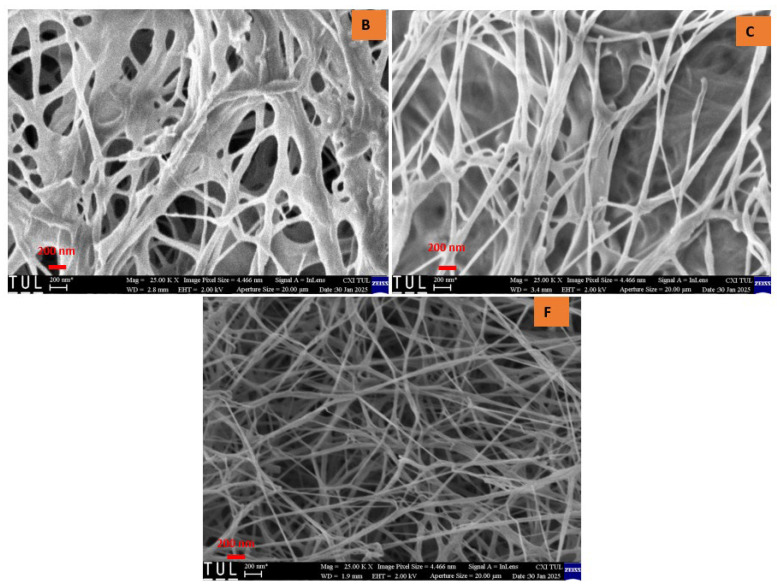
Cross-sectional area of BCAs. According to the sample description in Table 7. (B,C) conventional pre-freezing BCAs; (F) LN2 pre-freezing BCAs.

**Figure 7:**
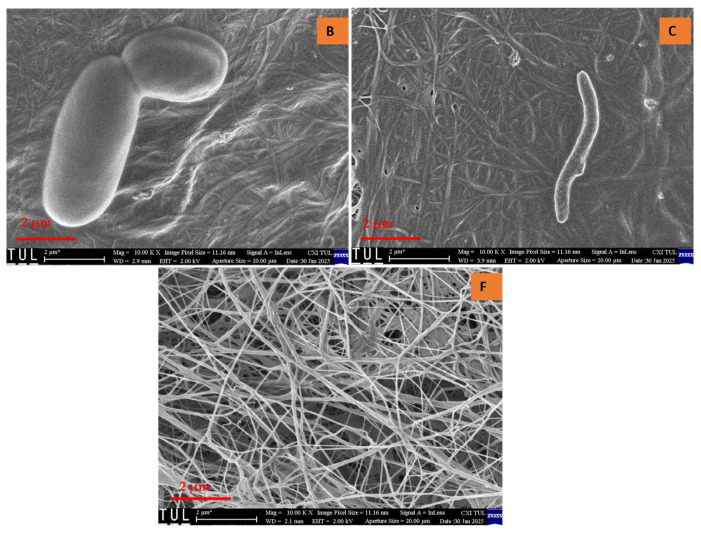
Surface of the BCAs. According to the sample description in Table 7. (B,C) conventional pre-freezing BCAs; (F) LN2 pre-freezing BCAs.

Upon examining the texture of all samples, a randomly arranged fibrous structure was observed. Consistent with previously reported findings, bacterial cellulose aerogels (BCAs) exhibited fine, densely packed fibers that interweave and crisscross, forming fibrils [21,22]. Structural analysis of the studied BC aerogels revealed a three-dimensional network with a porous configuration composed of ultrafine fibers arranged in an unpredictable, ribbon-like pattern. The rapid freezing of the BC aerogels using liquid nitrogen effectively preserved the integrity of the highly porous structure, resulting in aerogels that exhibit an interwoven, crisscrossed pattern, thereby creating a fibril-based three-dimensional arrangement, as depicted in Figure 6 and Figure 7. In contrast, the fiber arrangement in BCAs displayed a more compact structure, with reduced porosity between the fibers in both B and C. Furthermore, both fiber density and inter-fiber porosity were diminished, whereas an increase in fibrillation and porosity was observed in the BCA samples.

Contrary to the findings of Hamsan et al. and Maryati et al. [21,22], the traditional pre-freezing procedure was employed for samples B and C. This approach was chosen due to the frosting observed on the surface of BCA samples, which exhibited a foil-like, non-porous coating with minimal fibrillation. In contrast, the surface of the sample treated with LN_2_ pre-freezing displayed a randomly oriented fibrous structure, which was consistently visible. Additionally, fine and compact fibers interlaced to form crosswise fibrils, distinguishing them from the typical pre-freezing samples.

### 2.3. Surface Roughness Analysis

**Figure 8:**
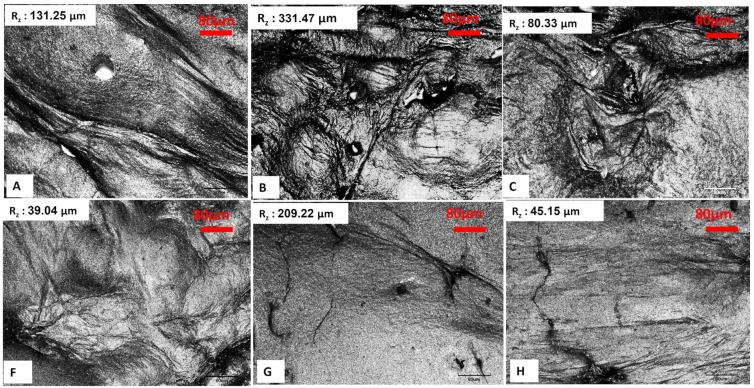
Lyophilized BCAs with conventional pre-freezing according to the sample description in Table 7 and (A–C) with LN_2_ pre-freezing (F–H). Objective: 20× for all images. Scale bar represents 80 µm. Rz: mean roughness depth.

A confocal scanning infrared (IR) laser microscope serves as an effective instrument for evaluating the surface roughness of bacterial cellulose aerogels (BCA), as it provides high-resolution three-dimensional surface topography. This technique facilitates the quantitative assessment of roughness parameters, which are essential for understanding surface properties in healthcare, food, and materials science applications. Laser-based surface texturing is an emerging and potent technology that has the potential to modify the surface roughness and chemistry of a wide range of materials [23,24,25,26]. Moreover, it facilitates the precise design of diverse micro and nano-topographic patterns, ensuring high spatial and temporal resolution, repeatability, and flexibility. In addition to its capability as a high-speed processing technology that is readily automatable, it also minimizes the risk of surface contamination due to the absence of direct contact during operation [27]. Surface evaluation of lyophilized BCAs, utilizing both conventional and LN_2_ pre-freezing methods, demonstrated that the BCAs subjected to LN_2_ pre-freezing with the greatest thickness exhibited the lowest surface roughness. This was followed by the thinnest BCAs subjected to LN_2_ pre-freezing, as indicated by the mean roughness index in Figure 8. Scanning electron microscopy (SEM) images of the BCAs surfaces revealed that those processed with LN_2,_ and standard pre-freezing techniques exhibited less fibrous structures (Figure 6 and Figure 7).

**Figure 9:**
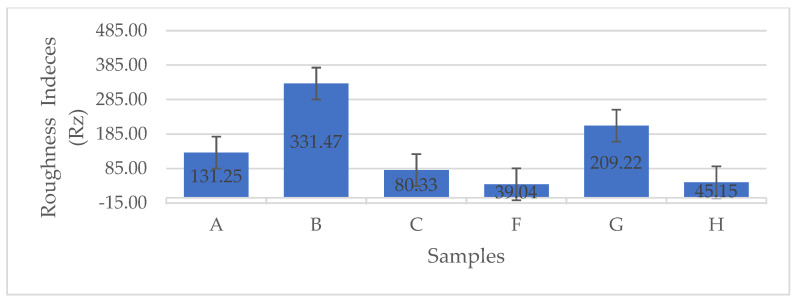
The error bar plots show the differences in the mean roughness indices (Rz, μm) between the different BCA surfaces [according to the sample description in Table 7]. (A−C) stabilized structure of BC with conventional pre-freezing used before lyophilization; (F−H) LN_2_/fast freezing used before lyophilization.

The error bar plot presented in Figure 9 illustrates the variations in mean roughness indices (R_z_, μm) among the BCA samples. As shown in Figure 9, the BCA samples subjected to LN_2_ pre-freezing and those utilizing a structural form exhibited the lowest roughness indices. These findings clearly indicate that the use of a PES NW fabric-covered Petri dish effectively insulated and facilitated uniform heat distribution, as well as consistent moisture removal from the sample. Consequently, this approach resulted in reduced roughness indices.

**Figure 10:**
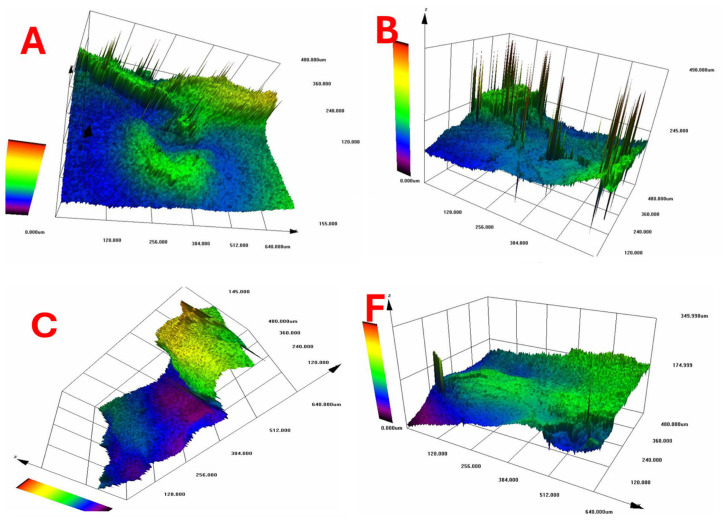

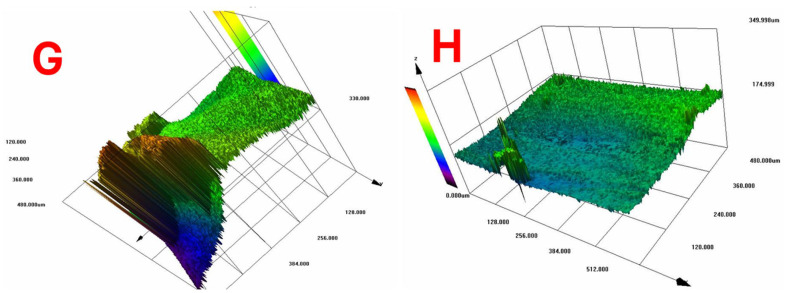
Surface topography of BC films. Conventional vs. LN_2_ pre-freezing according to the sample description in Table 7: (A–C) stabilized structure of BC with conventional pre-freezing used before lyophilization; (F–H) LN_2_/fast freezing used before lyophilization.

According to Figure 10, which shows the surface topography of BC films, conventional pre-freezing (A, B, C) resulted in higher surface roughness and irregular textures, likely due to slower ice crystal growth causing structural shrinkage. LN_2_ pre-freezing (F, G, H) resulted in mixed effects. Sample G showed extreme roughness, likely due to rapid ice formation creating surface stress. Sample H was the smoothest, suggesting that liquid nitrogen pre-freezing can lead to more uniform solidification under optimal conditions.

The findings suggest that LN_2_ pre-freezing can either increase or reduce roughness, depending on how it influences ice nucleation and cellulose network stabilization. The effect of LN_2_ pre-freezing on surface roughness is highly dependent on how the cellulose network responds to ultra-fast freezing. If the freezing rate is too fast and causes ice-induced stress, roughness increases (Sample G). However, if freezing happens in a controlled and uniform manner, roughness decreases, leading to a smoother surface (Sample H).

### 2.4. Water Contact Angle (WCA) Analysis

The BCA material exhibits a relatively low water contact angle (WCA) of approximately 30°, indicating its highly hydrophilic nature. Consequently, prolonged exposure to water leads to the degradation of the cellulose fiber structure. This deterioration occurs as water disrupts the hydrogen bonds between the fibers, resulting in material swelling and a subsequent reduction in mechanical strength [28,29]. The porosity and absorbency of BCA render it beneficial for specific applications, such as food additives and wound dressings. However, these properties also impose limitations on its applicability in certain industries, including filter membranes and textiles [30]. In this study, freeze–dried BCAs subjected to liquid nitrogen pre-freezing and conventional pre-freezing were analyzed using WCA tests. The findings revealed that both pre-freezing methods resulted in hydrophilic samples.

**Figure 11:**
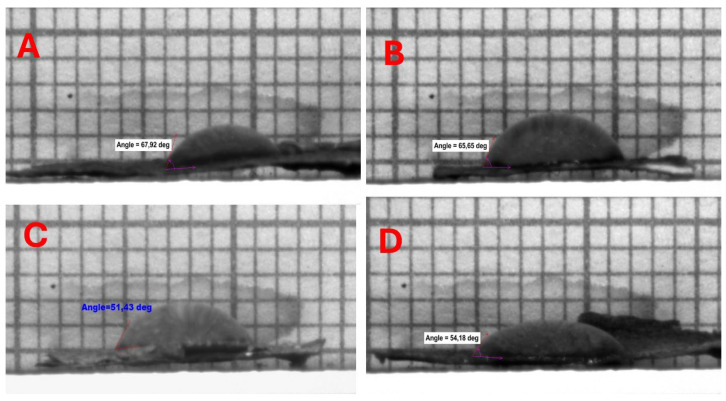
Hydrophilicity tests on BCAs frozen with a conventional freezer and stabilized structures placed between PES NW fabrics. (**A**) BCA placed in between NW (15 GSM) fabric; (**B**) the bottom of the Petri dish placed on NW fabric (10 GSM) and BCA placed on top; (**C**) top of Petri dish covered with NW fabric (10 GSM) and BCA placed on top; (**D**) bottom of the Petri dish placed on NW fabric (15 GSM) and BCA placed on top.

The water contact angle (WCA) of all bacterial cellulose (BC) samples subjected to liquid nitrogen (LN_2_) pre-freezing prior to drying was observed to be zero degrees, indicating a super hydrophilic nature. In contrast, BC samples that underwent conventional pre-freezing exhibited WCA values ranging between 51.18° ± 0.25 and 65.65° ± 2.28. This difference can be attributed to the formation of ice crystals on the surface of the samples during the freezing process, which led to the development of a foil-like structure on the bacterial cellulose surface, thereby obstructing the pores, as illustrated in Figure 11.

### 2.5. Analysis of Young’s Modulus Under Different Loads

BCA possesses various biorelevant features, including biological compatibility, high mechanical strength, and sorption capacity. This makes it a unique carrier matrix for enzymes, cells, and drugs, particularly those with hemostatic, antimicrobial, and regenerative properties [5,31]. Most practical uses of aerogels necessitate specific strength characteristics. As a result, identifying and understanding the factors influencing aerogel strength is critical. A BCA’s strength is determined by its microporous structure and density [32]. BCAs are very porous, lightweight, and compressible. When subjected to various loads, their thickness changes reveal important information about their mechanical behavior, structural robustness, and prospective uses (e.g., biomedical, packaging, thermal insulation).

**Figure 12:**
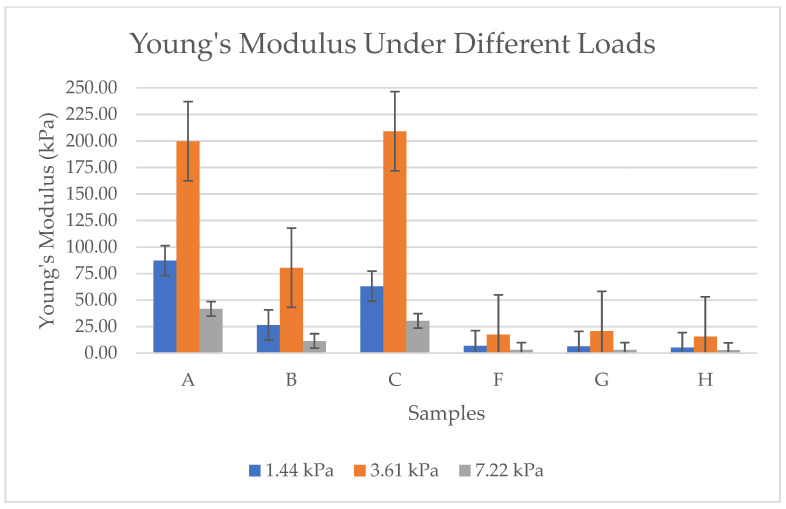
Young’s modulus under different loads for BCA samples according to the sample description in Table 7. (A–C) Conventional pre-freezing samples before lyophilization; (F–H) LN2 pre-freezing samples used before lyophilization.

As per the results in Figure 12, each sample has different values. Sample A and Sample C consistently show the highest Young’s modulus values across all loads. These values indicate superior structural integrity and resistance to deformation, making them suitable for applications requiring higher mechanical strength. Sample B has moderate stiffness compared to A and C; Sample B has lower Young’s modulus values. This means Sample B is softer and more flexible but still offers better resistance compared to the softer aerogels (F, G, H). Samples F, G, and H are the softest aerogels. These samples exhibit extremely low Young’s modulus values, indicating high compressibility and low resistance to deformation. Generally, all samples show an increase in Young’s modulus with higher loads, which suggests that bacterial cellulose aerogels stiffen under compression. The primary reason for the significant variation in these values is the morphological differences between the samples pre-frozen with liquid nitrogen (F, G, H) and those subjected to conventional pre-freezing (A, B, C). While the surface of the conventionally frozen samples displayed a foil-like structure due to ice crystal formation, the samples frozen with liquid nitrogen exhibited a softer, more porous structure with higher density. Thus, Samples A and C are the most mechanically robust, while F, G, and H are the softest. Sample B sits in between, balancing some stiffness with moderate flexibility.

### 2.6. Thermal Conductivity

The heat conduction capacity of a material is influenced by its thermal conductivity. The thermal conductivity of fabrics is calculated using the following Equation (4):

(1) $$q=-\lambda \;A\frac{\Delta T}{\Delta x}$$

In this equation, λ represents the material’s thermal conductivity, A denotes the cross-sectional area through which heat flows, and ΔT is the temperature gradient across two surfaces that are separated by a distance Δx.

Research on the thermal properties of BCA showed that freeze–drying enhanced the porous structure of BC aerogels and reduced thermal conductivity. Furthermore, depending on the composite structure employed, this method proves to be more eco-friendly and cost-efficient compared to supercritical CO_2_ drying [33,34]. Fan et al. observed that freeze–dried polyimide (PI) aerogels exhibit a thermal conductivity of 53 mW m^−1^ K^−1^, a result linked to the substantial pore sizes formed during the freezing process [35].

**Figure 13:**
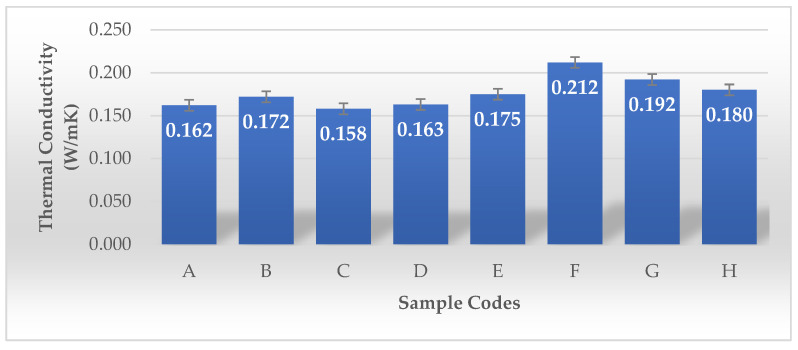
Thermal conductivity values of the BCAs according to the sample description in Table 7. (A–E) Conventional pre-freezing samples before lyophilization; (F–H) LN2 pre-freezing samples used before lyophilization.

Zang and colleagues developed a lightweight, bidirectionally anisotropic aerogel composed of polyimide and bacterial cellulose (b-PI/BC) through a bidirectional freezing technique. Bacterial cellulose, characterized by its ultra-fine nanofibrous structure, serves as a strengthening nanofiller within PI aerogels. Its incorporation inhibits shrinkage, maintains the aerogel’s structural stability, and leads to enhanced porosity while reducing density. These characteristics enhance thermal insulation by reducing heat transfer. In contrast to random and unidirectional freezing methods, the b-PI/BC aerogel produced through bidirectional freezing exhibits a highly organized lamellar porous structure. This parallel lamellar structure reduces heat transfer in the direction perpendicular to the lamellae while enhancing heat distribution within the plane, thereby preventing heat concentration. The thermal conductivity of the b-PI/BC aerogel is 23 mW m^−1^ K^−1^ in the radial direction (perpendicular to the lamellae), and it nearly doubles to 44 mW m^−1^ K^−1^ in the axial direction (parallel to the lamellae) [36]. As shown in Figure 13, the thermal conductivity of Samples A to E exhibits lower thermal conductivity (0.158–0.175 W/mK). Their porosity values are very high (98.81–99.87%), which likely contributes to lower thermal conductivity by reducing heat transfer through solid pathways. Sample E, despite having the highest volume density (360.36 g/cm^3^) among this group, still shows relatively low thermal conductivity (0.175 W/mK). This may be due to its rectangular shape or the plastic frame structure affecting heat dissipation. Samples F, G, and H show higher thermal conductivity (0.180–0.212 W/mK), with F having the highest (0.212 W/mK). These samples underwent LN_2_ pre-freezing, which likely altered their microstructure, leading to increased density and, consequently, higher thermal conductivity. Their volume density is significantly higher (93.60–181.63 g/cm^3^) than the non-LN_2_-treated samples. Despite their high porosity (~99.97–99.99%), the higher dry mass and structural arrangement could have contributed to greater solid-phase connectivity, enhancing heat transfer.

The fibrous materials are made up of trapped air and fibers. Stagnant air refers to the amount of air trapped in the fabric’s interior structure. Because stagnant air has a lower thermal conductivity value than fibers, the amount of stagnant air is an essential element influencing the thermal conductivity value of textile structures [37]. The thermal conductivity of air at room temperature (approximately 20 °C) is around 0.0264 W/m·K [38]. This value is significantly lower than the thermal conductivity values observed in our samples, which range from 0.158 to 0.212 W/m·K. The higher thermal conductivity in our samples is likely due to the solid matrix of the BC films, which facilitates more efficient heat transfer compared to air. Materials like Styrofoam have thermal conductivities around 0.033 W/m·K, which is higher than air but still lower than our samples [39].

Our samples are distinct from the low-density bacterial cellulose (BC) aerogels reported by Revin et al., which exhibit thermal conductivities of approximately 0.0257 W/m·K and densities between 4.2 and 22.8 kg/m^3^. In comparison, a study by Fleury et al. demonstrated that BC samples derived from brewery waste achieved thermal conductivities of about 13 mW/(m·K). Notably, Revin et al.’s samples were synthesized with the aid of supplementary chemicals, including peptone, disodium phosphate, and citric acid monohydrate [32]. Conversely, Fleury et al.’s BC samples utilized waste from the brewing industry as a source material [40]. In contrast, our samples were prepared exclusively from natural materials, without the incorporation of additional chemicals or waste materials. In summary, our BC aerogel samples exhibit higher thermal conductivities than those of air or Revin et al. and Fleury et al’s BC samples, primarily due to their solid structure, which enhances heat conduction.

## 3. Conclusions

The *Acetobacter xylinum* CCM 2360 strain was successfully cultivated using a static culture method, with tea extract, water, and sugar serving as the nutrient sources for bacterial cellulose (BC) film synthesis. BC aerogels (BCAs) were fabricated with different supporting structures, including nonwoven (NW) fabric, plastic frames, and liquid nitrogen (LN_2_) pre-freezing techniques to assess their impact on thickness, density, and porosity, which influence the aerogels’ mechanical and functional properties.

The Amaru lyophilization drying method was employed under optimized conditions (−30 °C pre-freezing, −40 °C drying for 3 h, 48 h total process, and 0.133 mBar vacuum pressure). The results indicate that LN_2_ pre-freezing significantly increases the dry mass and volume density compared to NW-supported aerogels, whereas plastic-framed samples exhibited the highest volume density. Despite this, porosity remained above 98% for all samples, confirming their lightweight nature. However, one of the key challenges remains the identification of factors contributing to crack formation and the development of methods to achieve a completely homogeneous and smooth surface structure.

SEM analysis revealed that LN_2_ pre-freezing preserved a three-dimensional porous network with ultrafine, ribbon-like fibers, whereas conventional pre-freezing produced non-porous, foil-like surfaces with reduced fibrillation. This suggests that LN_2_ pre-freezing enhances fibrillation and porosity, resulting in a denser yet highly porous structure.

Surface roughness analysis indicated that LN_2_-pre-frozen samples exhibited the lowest roughness, especially in the thickest BCAs. The incorporation of PES NW fabric-covered Petri dishes improved heat insulation and moisture removal, yielding smoother surfaces. Despite these improvements, achieving a completely uniform surface texture remains a limitation, necessitating further refinement of processing techniques.

Water contact angle (WCA) measurements demonstrated that LN_2_-pre-frozen samples were superhydrophilic (0° WCA), while conventionally pre-frozen samples had higher WCA values (51.18°–65.65°), likely due to ice crystal formation reducing pore exposure.

Mechanical testing showed that rigidity correlated with microporosity and density. Samples A and C exhibited the highest Young’s modulus, indicating superior stiffness, while B was moderately stiff. Conversely, LN_2_-pre-frozen samples (F, G, H) were the most compressible due to their highly porous nature.

Thermal conductivity analysis revealed that conventionally pre-frozen samples (A–E) exhibited lower values (0.158–0.175 W/mK) due to high porosity (98.81–99.87%), whereas LN_2_-pre-frozen samples (F–H) demonstrated higher values (0.180–0.212 W/mK) due to increased density and solid-phase connectivity. The elevated thermal conductivity in all samples is likely attributed to their purely natural composition and solid structural arrangement.

From the results, it can be concluded that the synthesized BC aerogels exhibit unique properties, making them highly versatile for wearable technology, packaging applications, thermal insulation, biomedical uses, and filtration. Nevertheless, addressing the challenge of crack formation and surface uniformity will be essential for optimizing their performance in advanced applications.

## 4. Materials and Methods

### 4.1. Materials

The *Acetobacter xylinum* (CCM 2360) strain was obtained from the Czech Collection of Microorganisms Department of Experimental Biology. (Brno, Czech Republic,) Polyester Nonwoven (PES NW) fabric (10, 15 GSM) was purchased from JX Nippon ANCI Corporation. (Aix-en-Provence, France). The black tea (PG Tips Loose Leaf catering black tea) as a nitrogen source and sugar (TTD cukr krupice bily) as a carbon source for fermentation processes were obtained from the market in the Liberec, Czech Republic. Sodium carbonate (Na_2_CO_3_ (molecular weight: 105.99)) without water was purchased from Chemapol, (Prague, Czech Republic) for purification of the BC aerogel. Liquid nitrogen (LN_2_) was obtained from LovoChemie, (Prague, Czech Republic) for pre-freezing to compare the conventional (refrigerator) freezer method.

### 4.2. Fabrication of BC Pellicles

The preparation of bacterial cellulose started with forming a sweet black tea solution. The sweet tea solution was prepared with 48% *w*/*v* sugar and 1.2% *w*/*v* tea leaves with water and boiled for approximately 20 min for extraction of tea leaves. The process continued with measuring the bacteria amount with a Densitometer McFarland type DEN. After the tea solution cooled down, one-tenth of the volume of *Acetobacter xylinum* bacteria was added to the tea solution to initiate fermentation. Optimal fermentation occurred at 28 °C with a pH range of 4. After four days, a 3 mm thick fungus layer formed, which hardened to 5 mm in seven days, depending on the desired thickness of BC. According to Table 5, the cultivation process of BC films is given for the experimental conditions (temperature, cell concentration, pH, duration of the samples to obtain thick pellicle structure) and cultivation parameters of *Acetobacter xylinum* pellicles in static mode.

**Figure 14:**
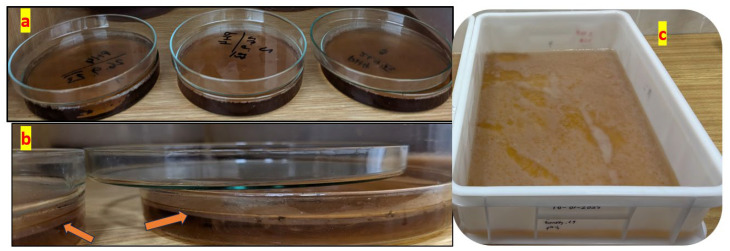
Standard preparation of BC samples in static mode. (**a**) BC film growth in Petri dish; (**b**) close-up growth of BC films at approximately 2–3 mm; (**c**) rectangular plastic box to have large samples in.

BC films were grown on a Petri dish, as shown in Figure 14a, and in a rectangular plastic box to have large samples in static mode, as shown in Figure 14c. The formation of BC films with a thickness of 2–3 mm on the tea solution’s surface is highlighted in the close-up in Figure 14b.

**Figure 15:**
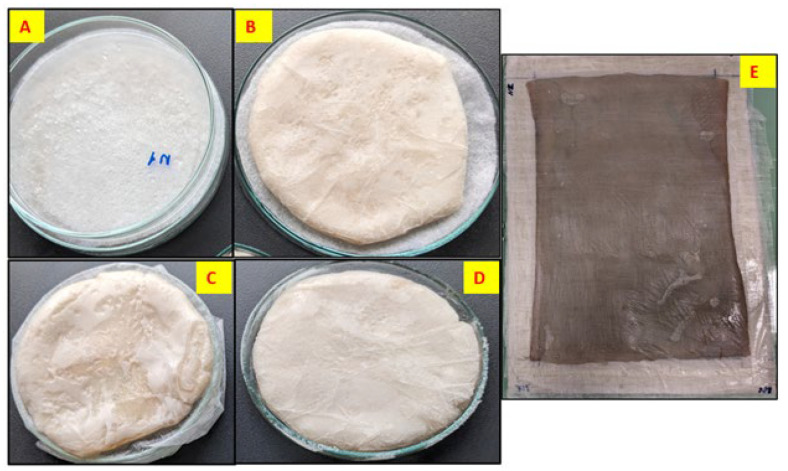
The form stabilization of BC films to enhance properties during lyophilization. According to the sample description in Table 7. (**A**–**E**) Conventional pre-freezing samples before lyophilization.

After the synthesis of BC films, the samples were stabilized with Polyester nonwoven (PET NW) fabrics in different forms to obtain a homogeneous-smooth surface structure on BC films. As in Figure 15, the BC films were prepared for form stabilization with the PET NW fabrics. Based on the fundamental physical and thermal properties of using nonwoven fabric placed in a Petri dish during lyophilization, as opposed to the fabric’s direct contact with the Petri dish surface, the use of NW fabrics is intended to improve the structural and physical properties of our bacterial cellulose samples by lowering stress, increasing moisture removal, and controlling heat transfer. In order to help the lyophilization process, PET NW textiles of varying thicknesses and weights were used in an effort to enhance the qualities of BC films (Table 6 depicts the properties of PET NW fabrics).

PET NW fabrics have been placed in Petri dish in different positions, like BC films placed in between PET NW (15 GSM) fabrics, A; the bottom of the Petri dish placed in PET NW fabric (10 GSM) with the BC film placed on it, B; the top of the Petri dish covered with PET NW fabric (10 GSM) and the BC films placed on it, C; the bottom of the Petri dish having a PET NW fabric (15 GSM) and BC film placed on it, D; and lastly, the BC films placed in a plastic frame structure with a PET NW fabric (10 GSM), E. Even though we would like to compare the thickest and thinnest BC film’s properties and structures, the BC samples’ thicknesses are slightly different from each other due to the uncontrollable growing process of BC films. Table 7 shows the description and properties of BC Film samples in wet conditions before lyophilized. Due to the LN_2_ sample’s dimensions, the dimensions of the BC film’s growth in the Petri dish are smaller and lower in weight in wet conditions.

Subsequently, BC film samples that had been prepared for form stabilization underwent two distinct freezing processes—conventional (slower) freezing and (rapid freezing) liquid nitrogen freezing (LN_2_)—before being dried by lyophilization. The freezer in the home refrigerator is referred to as the “conventional freezing method”. A new technique that would have less of an impact on the BC film structure was studied, and liquid nitrogen was used in comparison after it was discovered during the experiments that crystallization had taken place on the surface of BC films frozen using the conventional freezing method and that the surface structure had been harmed. Samples F, G, and H were frozen using liquid nitrogen, while samples A, B, C, D, and E were frozen using the traditional freezing method. Samples F, G, and H represent bacterial cellulose (BC) films subjected to LN_2_ pre-freezing but remain in their wet form before lyophilization. The primary distinction among these samples lies in their wet thickness and wet mass, which indicate variations in water retention and structural integrity before drying. Sample F exhibited the highest wet thickness (0.693 cm) and the greatest wet mass (210.11 g), suggesting that it retained more moisture and maintained a relatively thicker structure. Sample G, with a wet thickness of 0.524 cm and a comparable wet mass (207.22 g), was slightly thinner than Sample F but still holds a significant amount of water. In contrast, Sample H showed the lowest wet thickness (0.275 cm) and wet mass (104.96 g), indicating a more compact structure with reduced water content. These variations imply that different factors, such as initial hydration levels, film handling, or pre-freezing effects, may have influenced the physical properties of BC films at this stage. The BC film samples created for form stabilization were then frozen using two distinct methods before being dried: conventional freezing and liquid nitrogen freezing methods. The conventional freezing method refers to the freezer within the household refrigerator. However, during the trials, it was discovered that crystallization formed on the surface of BC films frozen using the conventional freezer method, deteriorating the surface structure, so a new method that would have a lower impact on the BC film structure was investigated, and liquid nitrogen was used as a comparison. Samples A, B, C, D, and E were frozen for 24 h using the conventional freezing method; samples F, G, and H were frozen using liquid nitrogen. The samples were then pre-frozen at −30 degrees for 3 h using a fully automatic Lyotrade-Amaru Freeze Dryer (Prague, Czech Republic) with heated shelves (30–40 mild-heating) at −30 °C pre-freezing temperature, followed by 48 h of drying at −40 degrees and 0.133 mbar vacuum pressure.

### 4.3. Morphology

The morphological structure of BC was examined by scanning electron microscopy (SEM) with a Zeiss Ultra Plus Model SEM, Potsdam, Germany; the accelerating voltage was controlled at 2 kV.

### 4.4. Surface Roughness

The surface roughness structure of the bacterial cellulose specimens was studied using a confocal scanning infrared (IR) laser microscope, Olympus corporation (Hachioji-shi, Tokyo, Japan) by LEXT-OLS3100 Software MM6-ASPS equipped with a 405 nm laser, 20× magnification lenses with ČSN EN ISO 21920-2 (014450) standard [41].

### 4.5. Characterization of Water Contact Angle

A droplet shape analyzer (DSA) with NIS Elements AR Software Microscope version 6.10.01, Naviter Camera, by Laboratory Imaging s.r.o. Nikon Corporation, (Prague, Czech Republic) and 0.5 lenses were used to deposit 0.05 mL droplets on BC surfaces in order to measure the hydrophilicity of dried samples using water contact angle (WCA) tests.

### 4.6. Young’s Modulus Under Different Loads

**Figure 16:**
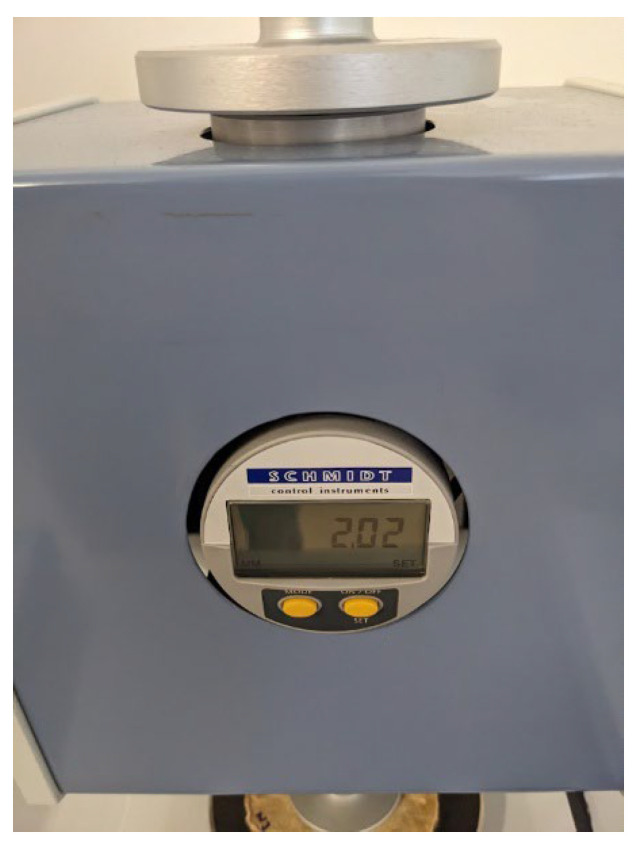
Thickness test measurement machine with different loads.

The modulus of elasticity of the BC aerogels under compressive loads was determined by the compression test under various loads. Measurements were taken in accordance with ASTM 1777 Version 2 [42] using a compression test measuring device of the type of Schmidt Control Instrument Thickness Gauge D-2000 (Waldkraiburg, Germany). Thickness measurements were performed at pressures of 1 kPa, 100 g, 200 g, and 500 g. Figure 16 shows the testing machine, which includes a sample and a pressure unit.

### 4.7. Thermal Conductivity (Alambeta)

The thermal conductivity coefficient of BC aerogels under varying pre-freezing conditions was investigated using the Alambeta device (Sensora, Liberec, Czech Republic). The measurement process is based on assessing heat flux across the sample, which is subjected to a temperature gradient of 32 °C between the device’s upper and lower plates. The experiment was conducted under ambient temperature conditions, where heat transfer occurs from the upper plate to the lower plate through the sample placed in between [43].

### 4.8. Porosity Determination

To assess the porosity, the dimensions and mass of each BC sample were recorded. The apparent total volume $V_{a\;}$ [m^3^] of the sample was then calculated by multiplying the average thickness h [m] by the surface area A [m^2^] of the BCA sample.

(2) $$V_{a\;}\left[m^{3}\right]=hA$$

The cellulose content in the sample, denoted as $V_{c\;}$ [m^3^] was calculated by considering the sample’s mass, M [kg], along with the known density of cellulose, *ρ* [1560 kg/m^3^].

(3) Vc m3=Mρ

The porosity of BCA, denoted as P [%], is subsequently determined using the following equation [42]:

(4) P=100 1−VcVa

### 4.9. Statistical Analysis

Statistical analysis was presented with a one-way analysis of variance (ANOVA) and obtained from at least three independent tests.

## Figures and Tables

**Table 1:** Geometrical properties of BC films after lyophilization under different preparation conditions.

Samples	Description	After Lyophilization							
		Dry Thickness (cm)	Dry Mass (g)	Volume Density (g/cm^3^)	Porosity (%)	Surface Area (cm^2^)	Dimension (cm)	Skeletal Density (g/cm^3^)	Shape
A	BC film placed between NW (15 GSM) fabrics	0.45	0.592	31.88	98.81	70.85	4.75	1.24	Circular
B	Bottom of the Petri dish placed on NW fabric (10 GSM), and BC film placed on it	0.52	0.99	61.36	98.97	117.99	6.13	1.01	Circular
C	Top of the Petri dish covered with NW fabric (10 GSM), and BC film placed on it	0.56	0.35	48.47	99.54	86.55	5.25	1.12	Circular
D	Bottom of the Petri dish placed on NW fabric (15 GSM), and BC film placed on it	0.50	0.39	34.68	99.28	69.36	4.70	1.26	Circular
E	BC film placed in plastic frame structure	0.52	0.708	360.36	99.87	693.00	21 × 33	1.31	Rectangular
F	LN_2_ pre-freezing using BC film	0.72	2.92	181.63	99.99	252.96	13.6 × 18.6	0.22	Rectangular
G	LN_2_ pre-freezing using BC film	0.49	3.68	154.35	99.98	315.00	14 × 22.5	0.23	Rectangular
H	LN_2_ pre-freezing using BC film	0.27	3.85	93.60	99.97	344.10	15.5 × 22.2	0.51	Rectangular

**Table 2:** Comparison of pre-freezing conditions on porosity characteristics by one-way ANOVA.

Porosity of BCAs used Conventional Pre-Freezing						
Source of Variation	SS (Sum of Squares)	Df (Degrees of Freedom)	MS (Mean Square)	F (F-statistic)	*p*-Value (Probability)	*F crit* (Critical Value)
Between Groups	0.00989956	4	0.002475	103.1738	4.69 × 10^−13^	2.866081
Within Groups	0.00047975	20	2.4 × 10^−5^			
Total	0.01037932	24				
Porosity of BCAs using Liquid Nitrogen Pre-Freezing						
Source of Variation	SS	df	MS	F	*p*-Value	*F crit*
Between Groups	0.000676	2	0.000338	23.49707	7.08 × 10^−5^	3.885293835
Within Groups	0.000173	12	1.44 × 10^−5^			
Total	0.000849	14				

**Table 3:** Comparison of pre-freezing conditions on volume density characteristics by one-way ANOVA.

Volume density of BCAs using Liquid Nitrogen Pre-Freezing						
Source of Variation	SS	df	MS	F	*p*-Value	*F crit*
Between Groups	20,307.3	2	10,153.65	10.16171	0.002618	3.885294
Within Groups	11,990.48	12	999.207			
Total	32,297.79	14				
Volume Density of BCAs using Conventional Pre-Freezing						
Source of Variation	SS	df	MS	F	*p*-Value	*F crit*
Between Groups	15,282.12	4	3820.53	297.5808	1.64 × 10^−17^	2.866081
Within Groups	256.7726	20	12.83863			
Total	15,538.89	24				

**Table 4:** Comparison of pre-freezing conditions on thickness characteristics by one-way ANOVA.

Thicknesses of BCAs using Liquid Nitrogen Pre-Freezing						
Source of Variation	SS	df	MS	F	*p*-Value	*F crit*
Between Groups	0.497373	2	0.248687	23.46839	7.12 × 10^−05^	3.885294
Within Groups	0.12716	12	0.010597			
Total	0.624533	14				
Thicknesses of BCAs using Conventional Pre-Freezing						
Source of Variation	SS	df	MS	F	*p*-Value	*F crit*
Between Groups	2.935348	4	0.733837	279.3617	3.06 × 10^−17^	2.866081
Within Groups	0.052537	20	0.002627			
Total	2.987884	24				

**Table 5:** Experimental conditions and cultivation parameters of BC films.

Experimental Conditions	*Acetobacter xylinum* Cultivation Parameters
Cell concentration (inoculum)	1.3 × 10^9^ cells/mL (by McFarland device)
Culture method	Without agitated culture
Growing temperature	28 ± 3 °C
Initial pH (tea medium for growing bacteria)	5 (pH indicator paper)
Final pH of bacteria medium	4
Duration of experiment	10 days

**Table 6:** Polyester nonwoven fabrics’ properties.

Materials	Structure	GSM (g/m^2^)	Thickness (mm)	Density (g/m^3^)	Air Permeability (mm/s)
PET Nonwoven fabric	Two-direction PET filaments fabricated via thermal bonding	10	0.068 ± 0.002	0.15	6827.5
		15	2.14 ± 0.002	0.01	4035

**Table 7:** The description and properties of BC film samples in wet conditions before lyophilization.

Samples	Description	Before Lyophilization	
		Wet Thickness (cm)	Wet Mass (g)
A	BC film placed in between PET NW (15 GSM) fabrics	5.5	30.92
B	The bottom of the Petri dish is placed on PET NW fabric (10 GSM), and BC film is placed on top of it	5.72	64.26
C	Top of Petri dish is covered with PET NW fabric (10 GSM), and BC film is placed on it	4.66	22.1
D	The bottom of the Petri dish is placed on PET NW fabric (15 GSM), and BC Film is placed on it	4.64	26.75
E	BC Film placed in plastic frame structure with PET NW fabric (10 GSM)	5.12	39.26
F	LN_2_ pre-freezing used BC film (highest wet thickness and wet mass, indicating greater water retention before drying)	0.693	210.11
G	LN_2_ pre-freezing used BC film (slightly lower wet thickness than Sample F but similar wet mass, suggesting a denser structure or different hydration distribution)	0.524	207.22
H	LN_2_ pre-freezing used BC film (lowest wet thickness and significantly reduced wet mass, implying a more compact structure with lower water retention in the wet state)	0.275	104.96

## Data Availability

The original contributions presented in this study are included in the article. Further inquiries can be directed to the corresponding authors.
